# Incidence and risk factors for acute kidney injury in children with nephrotic syndrome: a meta-analysis

**DOI:** 10.3389/fped.2024.1452568

**Published:** 2024-12-20

**Authors:** Changdi Chen, Bingbing Qiu, Jianxin Wang, Liuqing Yang, Yanru Huang

**Affiliations:** Department of Pediatrics, The First Hospital of Quanzhou Affiliated to Fujian Medical University, Quanzhou, Fujian, China

**Keywords:** nephrotic syndrome, acute kidney injury, incidence, risk factors, meta-analysis

## Abstract

**Background:**

Nephrotic syndrome (NS) is a prevalent kidney disease in children. Acute kidney injury (AKI) is a severe complication of NS and has the potential to be life-threatening.

**Objective:**

The aim of this study was to analyze the prevalence and risk factors of AKI in children with NS, and to provide an evidence-based medical basis for the early identification of high-risk children in the clinic.

**Methods:**

A comprehensive search was conducted in publicly available databases, namely PubMed, Embase, Web of Science, Scopus, and the Cochrane Library, covering the period from the inception of each database until May 2024. The analysis involved examining basic characteristics (age, sex), the concomitant diseases (hypertension, infections), NS disease characteristics (steroid susceptibility classification, pathologic classification), laboratory test (e.g., serum albumin), and the use of nephrotoxic drugs. Traditional and network meta-analyses were performed for analysis.

**Results:**

A total of 11 studies were included in the analysis, revealing an incidence of AKI of 29% (95% CI: 23%–37%). The analysis of factors indicated that the age of NS onset [standardized mean difference (SMD): 0.31; 95% confidence interval (CI): 0.08, 0.54; *p* = 0.009], sex [odds ratio (OR): 1.49; 95% CI: 1.03, 2.16; *p* = 0.035], serum albumin level (SMD: −0.43; 95% CI: −0.85, −0.02; *p* = 0.041), response to steroid treatment (OR: 0.52; 95% CI: 0.33, 0.80; *p* = 0.003), infection (OR: 3.60; 95% CI: 1.91, 6.78; *p* < 0.001), hypertension (OR: 4.02; 95% CI: 2.94, 5.51; *p* < 0.001), and nephrotoxic drug application (OR: 4.43; 95% CI: 1.86, 10.53; *p* = 0.001), were all significantly associated with the incidence of AKI. Furthermore, the results of the network meta-analysis suggested that the pathologic type of minor glomerular abnormalities (MGA)/diffuse mesangial proliferation (DMP), the type of infrequent relapses (IFRNS)/steroid-sensitive NS (SSNS), and the use of diuretic medications were associated with a relatively low risk of AKI occurrence.

**Conclusion:**

Factors upon admission of children with NS are associated with the onset of AKI. Emphasis should be placed on populations with a heightened risk of AKI in clinical practice. Further research is warranted to confirm the findings due to the limitations of this study.

**Systematic Review Registration:**

https://www.crd.york.ac.uk/prospero/display_record.php?ID=CRD42024571170, PROSPERO (CRD42024571170).

## 1 Introduction

Nephrotic syndrome (NS) is one of the most common types of kidney disease in children, and it is characterized by significant proteinuria, hypoalbuminemia, generalized edema, and hyperlipidemia (1). The global incidence of NS is relatively stable, with approximately 2.92 (range 2–7) new cases per 100,000 children per year, and a prevalence of approximately 16 cases per 100,000 children (2, 3). The main complications of NS include infection, acute kidney injury (AKI), and thromboembolism (TE). Failure to timely diagnose and treat these complications may pose a threat to the patient's life (2).

AKI is a severe complication of nephrotic syndrome (NS), and its risk factors are intertwined with NS, resulting in increased hospitalization and mortality rates. Based on the diversity of diagnostic criteria for AKI, the incidence of AKI among hospitalized children with NS ranges from 3.3% to 58.6% (4, 5). Research also indicates that the onset of AKI significantly increases the patient's risk of long-term development of chronic kidney disease (CKD), end-stage renal disease (ESKD), and death (6). The causes of AKI in children with NS are multifaceted. Possible etiologies encompass both direct and indirect factors, include intravascular volume deficiency, acute tubular necrosis, interstitial nephritis, nephrotoxic drug usage, and bilateral renal vein thrombosis et al. However, in clinical practice, only a few methods have been proven effective in preventing or reducing the occurrence of AKI (7–9). Therefore, having a deep understanding of the progression pattern of kidney disease after the onset of AKI is particularly important. The purpose of this study was to analyze the incidence and risk factors of AKI in children with NS, and provide an evidence-based medical basis for the early identification of high-risk children in clinical practice.

## 2 Methods

This meta-analysis was conducted in accordance with the Preferred Reporting Items for Systematic reviews and Meta-Analyses (PRISMA) statement and Meta-analysis of Observational Studies in Epidemiology (MOOSE) reporting guidelines.

### 2.1 Literature search

Relevant studies were searched in databases including PubMed, Embase, Web of Science, Scopus, and the Cochrane Library, from database inception to May 2024. The literature search formula was performed using medical subject headings (MeSH), keywords and Boolean, as (“Children” OR “paediatric” OR “pediatric”) AND (“Acute Kidney Injury”) AND (“nephrotic syndrome”) without any language restrictions. Furthermore, we screened the references of relevant reviews to avoid omission.

The inclusion criteria for studies were as follows: (1) they were observational or cohort studies involving children with nephrotic syndrome; (2) they utilized clear definitions of AKI to classify children; and (3) they provided data on risk factors for AKI in NS patients. The predefined data included basic patient characteristics (age, sex), presence of concomitant diseases (hypertension, infection), NS disease characteristics (steroids-sensitivity classification, pathological classification), laboratory test results (such as serum albumin), and details of nephrotoxic drug usage. However, the time point of renal function tests was not fully stated in the included studies. Since there was a significant difference between the results of renal function tests at admission and when AKI occurred, the differences in renal function results were not analyzed in this study.

Studies were excluded based on the following criteria: (1) studies involving adult NS patients; (2) studies that lacked information on the diagnosis, classification, or characteristics of AKI patients; (3) studies that did not report the characteristics of the non-AKI group within the NS population; (4) studies that used Propensity Score Matching to select the control group (as it could result in inappropriate matching of key risk factors between groups); (5) studies that did not report the aforementioned risk factors of interest; (6) studies that were duplicates or had unextractable data. Furthermore, reviews, comments, and case reports were also excluded.

### 2.2 Literature screening and data extraction

Two authors rigorously screened all retrieved literature independently, adhering to the predefined inclusion and exclusion criteria. Initially, a preliminary screening was conducted by reviewing the titles and abstracts of all retrieved studies. Following the exclusion of duplicate studies and those not meeting the inclusion criteria, the remaining studies were identified through a comprehensive reading of the full text. The information such as the name of first author, country, year of publication, study design, sample size, AKI incidence, and reported risk factors was extracted from each eligible study. The outcome encompassed the original information on the risk factors between the AKI and no-AKI groups, as well as the unadjusted and multivariate-adjusted odds ratios (ORs) predicting the incidence of AKI. Any disputes that arose during the literature screening and data extraction process were resolved through discussion. In cases where consensus could not be reached, a third researcher (corresponding author) was consulted, and the final decision was determined by the corresponding author.

### 2.3 Design quality assessment

The risk of bias in the included studies was evaluated using the Newcastle-Ottawa Scale (NOS). This scale comprises three components: the representativeness of the exposed cohort, the comparability of the groups based on design or analysis, and the reported outcomes. Each study was assigned a total score out of 9 points, with ratings of 0–3, 4–6, and 7–9 indicating low-quality, moderate-quality, and high-quality studies, respectively. To ensure reliability, two independent reviewers conducted the quality assessments.

### 2.4 Statistical analysis

Meta-analysis was performed using R software (version 4.3.2). First, the incidence of AKI in each included study was pooled and analyzed. And then, the association between risk factors and the incidence of AKI was examined. For the pooled analysis, the original dichotomous and continuous outcome data were combined as odds ratios (OR) or standardized mean differences (SMD) with 95% confidence intervals (CI). Additionally, adjusted or unadjusted odds ratios (OR) values obtained from regression analysis were also utilized. The level of heterogeneity was assessed using the *I*^2^ statistic. If there was statistical heterogeneity (*p* < 0.05 or *I*^2^ ≥ 50%), a random effects model was employed for the pooled effect size analysis. Conversely, if there was no statistical heterogeneity (*p* ≥ 0.05 and *I*^2^ < 50%), a fixed effects model was used. Furthermore, we employed the formula proposed by Luo et al. (2018) to convert the median and interquartile range (IQR) of continuous data into mean and standard deviation (SD). To assess the influence of individual studies on the combined results, sensitivity analysis was conducted. Publication bias was examined through a visual funnel plot and evaluated using Begg's test, and Egger's test (for OR results) or Pustejovsky's test (for SMD results) (10). A significance level of *p* < 0.05 was considered indicative of a statistically significant difference. While network meta-analysis is primarily designed for indirect comparisons among high-quality studies, in this study, we also attempted to compare multiple categories in single risk factor type using the frequentist network meta-analysis. The risk of AKI in different risk factor arms was ranked using Surface Under the Cumulative Ranking curve (SCURA).

## 3 Results

**Figure 1:**
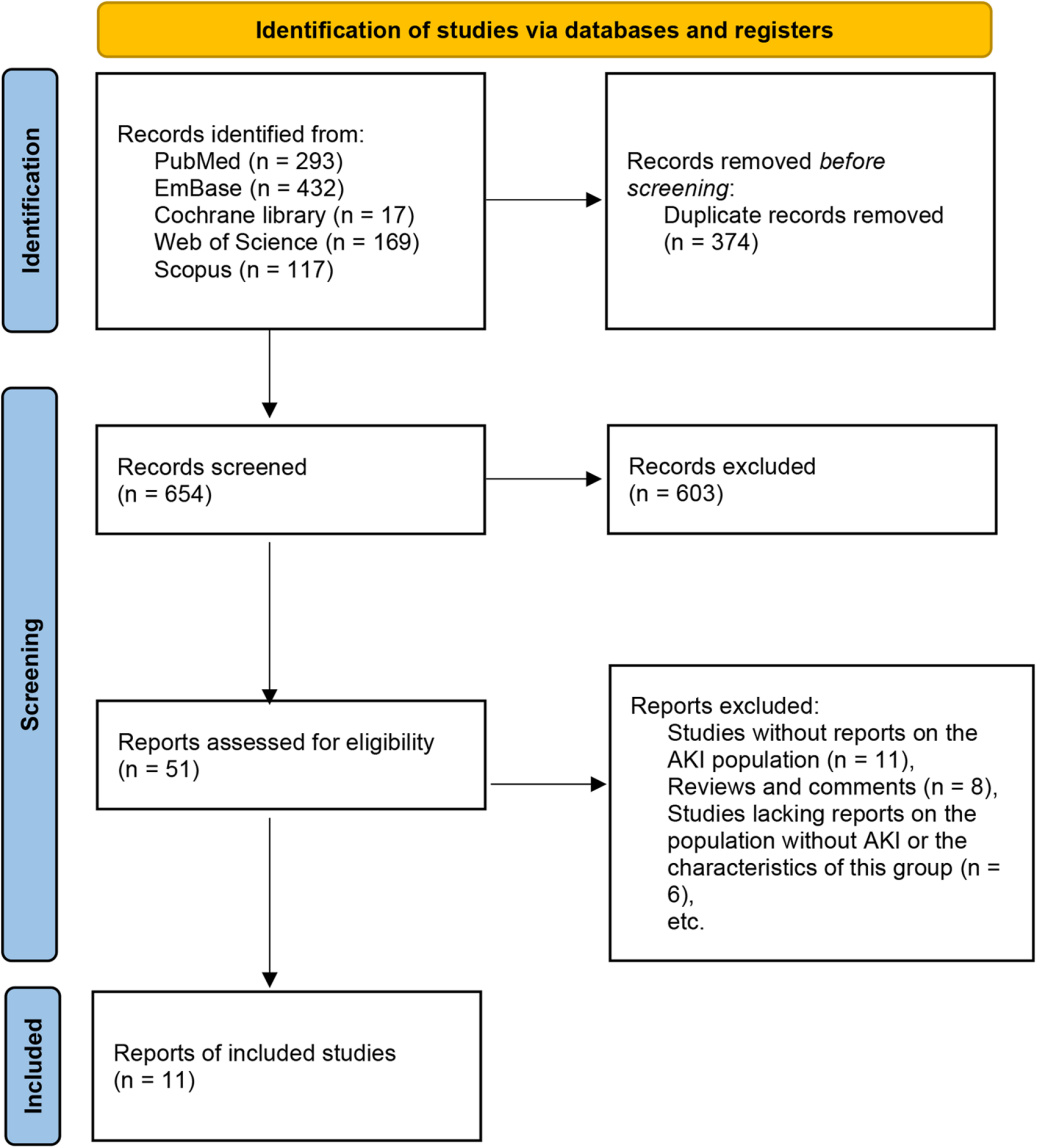
Flowchart of literature search and selection process.

A total of 1,028 articles were initially retrieved. After removing duplicates, 654 articles remained. Following the screening of titles and abstracts, 603 irrelevant studies were excluded. Subsequently, 51 studies underwent full-text review. The following articles were excluded due to: studies without reports on the AKI population (*n* = 11), reviews and comments (*n* = 8), studies lacking reports on the population without AKI or the characteristics of this group (*n* = 6), studies involving adult patients (*n* = 5), studies that included other types of patients without separate report on NS patients (*n* = 5), and case reports (*n* = 5). Ultimately, 11 studies were included for analysis (6, 11–20) (Figure 1, Table 1).

**Table 1:** The characteristics of included studies.

Study	Country	Design	Duration	Sample size	AKI (%)	Diagnosis	Factors
Yu et al. (2024) (6)	China	Retrospective	2014–2019	172	67 (39%)	KDIGO	Patient characteristics, NS type, lab test, infections, nephrotoxic drugs.
Ghosh et al. (2023) (11)	India	Prospective cohort study	2020–2021	200	36 (18%)	KDIGO	Patient characteristics, nephrotoxic drugs, lab test.
Ishiwa et al. (2022) (12)	Japan	Retrospective cohort	2002–2018	62	16 (25.8%)	KDIGO	Patient characteristics.
Anigilaje and Ibraheem (2022) (13)	Nigeria	Retrospective	2016–2021	75	19 (25.3%)	pRIFLE	Patient characteristics, lab test.
Yang et al. (2020) (14)	Korea	Retrospective multicenter study	2013–2017	363	89 (24.5%)	KDIGO	Patient characteristics, NS type, lab test.
Sato et al. (2021) (15)	Japan	Nationwide retrospective cohort	2010–2012	999	240 (24%)	KDIGO	Patient characteristics, serum albumin, concomitant diseases.
Kumar et al. (2021) (16)	India	Prospective observational study	2017–2018	54	23 (42.6%)	KDIGO	Patient characteristics, NS type, nephrotoxic drugs.
Prasad et al. (2019) (17)	India	Retrospective Observational study	2016–2017	73	13 (16%)	KDIGO	Patient characteristics, NS type, nephrotoxic drugs, lab test.
Sharma et al. (2018) (18)	India	Retrospective study	2012–1015	355	84 (23.7%)	pRIFLE	Patient characteristics, nephrotoxic drugs, infections.
Kim et al. (2018) (19)	Korea	Retrospective study	2015–2017	65	29 (32.2%)	KDIGO	Patient characteristics, nephrotoxic drugs, lab test.
Rheault et al. (2015) (20)	North America	Multicenter retrospective study	2010–2012	366	197 (58.6%)	pRIFLE	Patient characteristics, NS type, infections, lab test.

AKI, acute kidney injury; KDIGO, Kidney Disease Improving Global Outcomes; NS, nephrotic syndrome; pRIFLE, Pediatric Risk, Injury, Failure, Loss, End Stage Renal Disease criteria.

The included studies spanned from 2015 to 2024 and primarily focused on research conducted in Asia, specifically China, Japan, South Korea, and India. Additionally, two studies were conducted in Nigeria and North America (13, 20). Out of the total studies, two were prospective, while the remaining were retrospective. One study had an unclear type but leaned towards being retrospective based on its research content (17). The duration of the studies ranged mostly from 1 to 5 years. In total, 2,784 children with NS were included, with 813 of them having AKI. Only 8 children with secondary NS were included from the study by Anigilaje and Ibraheem (2022) (13), representing 0.3% of the sample, with the remaining 2,776 children having idiopathic NS. It is worth noting that some studies did not provide the exact number of patients with AKI but instead reported the frequency of AKI during hospitalizations. The quality of the included studies may have been influenced by certain factors, such as an unclear follow-up duration and the use of questionnaires instead of clinical records to gather information on AKI patients. However, the overall design quality of the studies was high (Supplementary Table 1).

**Figure 2:**
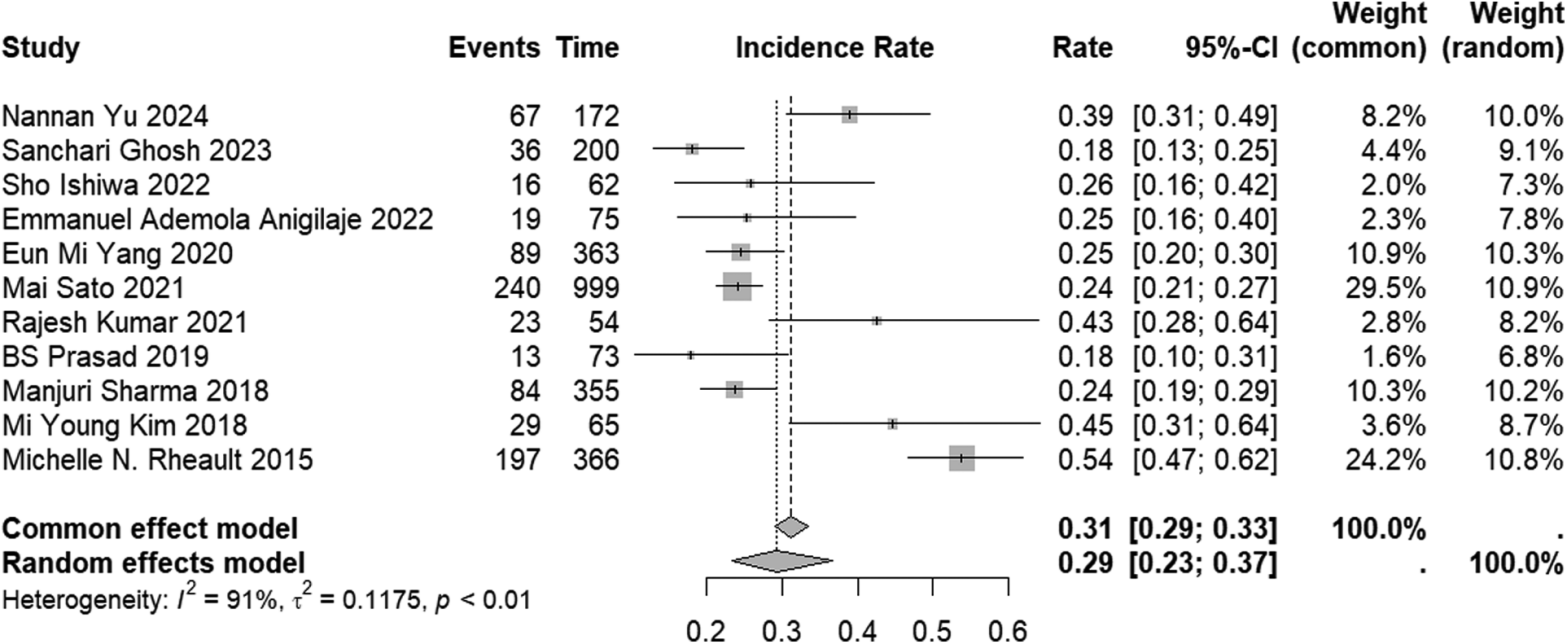
Forest plot of the incidence of AKI in children with NS.

The incidence of AKI in the included studies varied from 18% to 54%. When the results were combined, the overall incidence of AKI was found to be 29% (95% CI: 23%–37%) (Figure 2). However, studies that solely reported the incidence without providing information on the characteristics of risk factors for AKI occurrence were excluded. Therefore, it is important to interpret this result with caution. The subgroup analyses of AKI incidence across all subtype populations are presented in Supplementary Table 2.

**Figure 3:**
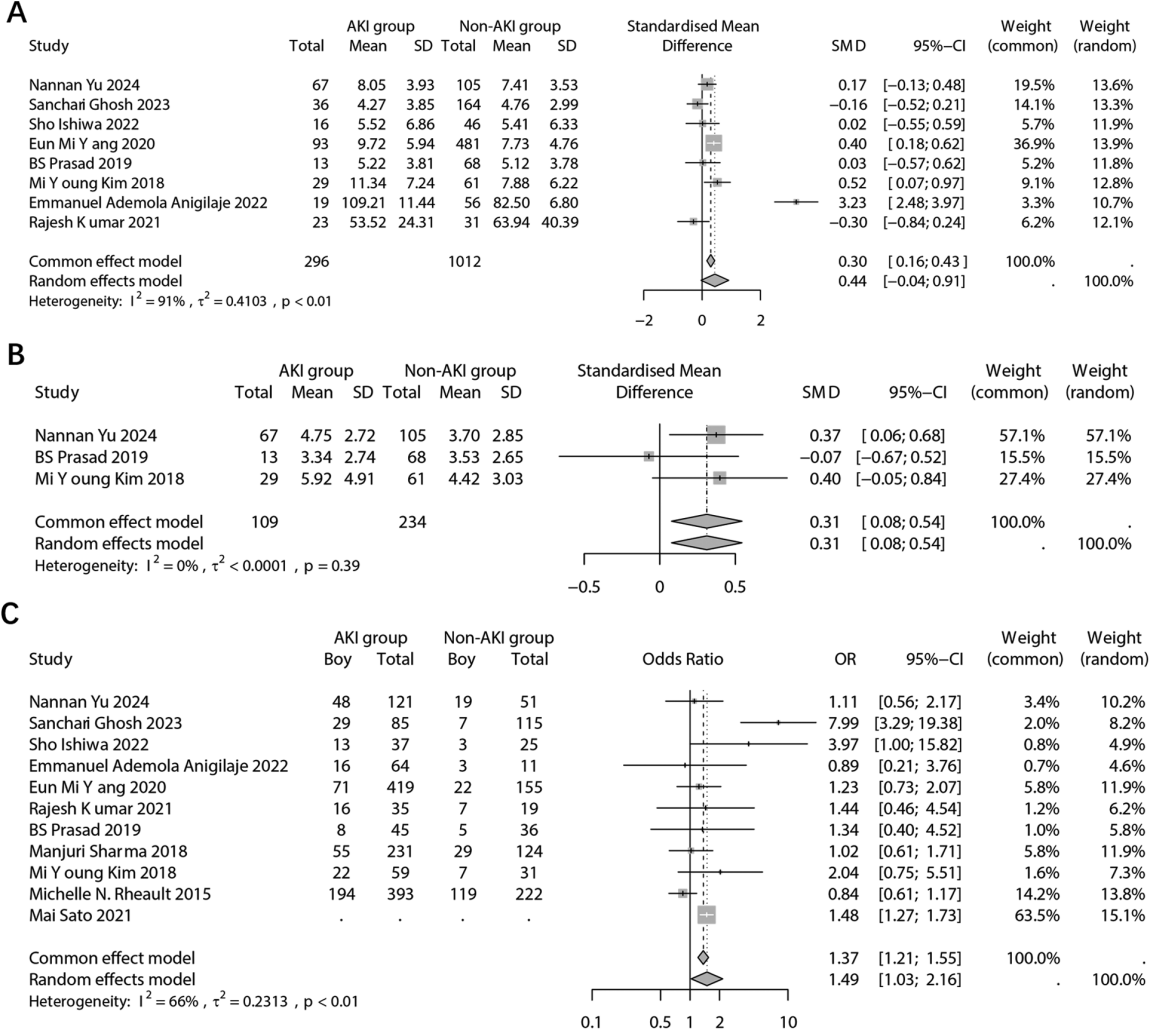
Forest plots showing the pooled results of age in admission **(A)**, age of NS onset **(B)**, and sex **(C)** between the AKI group and the non-AKI group.

For age, the analysis involved two aspects: the patient's admission age and the patient's age at the onset of NS. In the combined analysis of children's admission results using a random effects model, no significant difference in admission age was found between the AKI and non-AKI groups (SMD: 0.44; 95% CI: −0.04, 0.91; *p* = 0.071) (Figure 3A). Sensitivity analysis indicated that the findings reported by RajeshKumar 2021 significantly influenced the statistical difference in the combined results (Supplementary Figure 1A). Furthermore, the analysis of publication bias revealed no evidence of bias (Begg's test, *p* = 0.805; Pustejovsky's test, *p* = 0.997) (Supplementary Figure 1B).

A pooled analysis was also conducted on the age of onset of NS. The results, indicated a significant difference in the age of onset of NS between the two groups based on fixed effects model (SMD: 0.31; 95% CI: 0.08, 0.54; *p* = 0.009) (Figure 3B). The age of onset of NS in children with AKI was found to be significantly older. However, due to the limited number of included studies, sensitivity and publication bias analyses were not performed.

For children's sex. the results, using a random effects model, revealed a significant difference in sex distribution between the two groups (OR: 1.49; 95% CI: 1.03, 2.16; *p* = 0.035) (Figure 3C). Specifically, the proportion of boy was significantly higher in the group with AKI. It is important to note that the Mai 2021 study did not report the original frequency data but the OR value. Sensitivity analysis indicated that multiple studies may have influenced the final pooled results (Supplementary Figure 2A). Furthermore, the analysis of publication bias showed no evidence of potential bias (Begg's *p* = 0.484; Egger's *p* = 0.664) (Supplementary Figure 2B).

**Figure 4:**
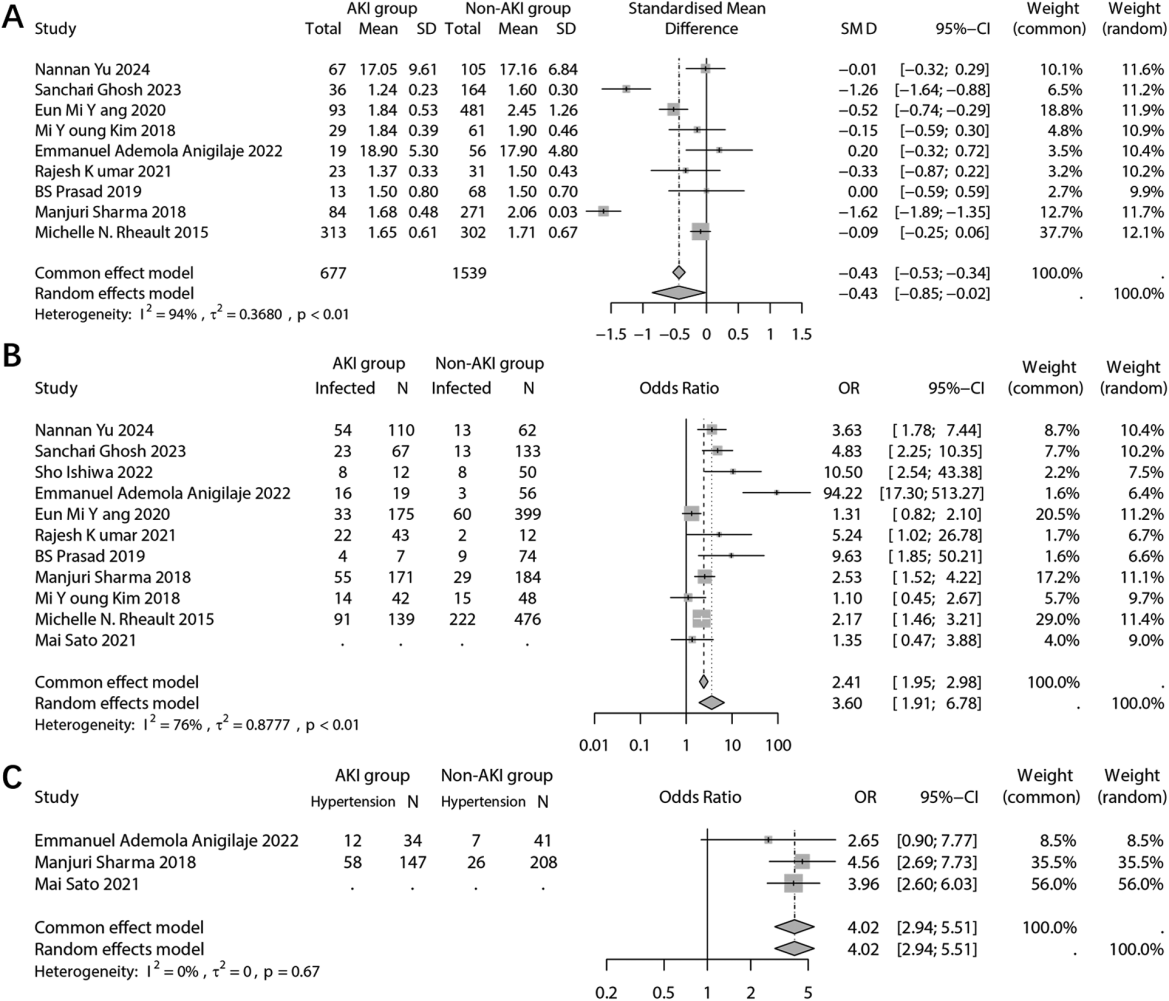
Forest plots showing the pooled results of serum albumin level **(A)**, infection **(B)**, and hypertension **(C)** between the AKI group and the non-AKI group.

In the laboratory test results, the analysis focused on the difference in serum albumin levels between the two groups. The combined results indicated a significantly lower serum albumin level in the population with AKI (SMD: −0.43; 95% CI: −0.85, −0.02; *p* = 0.041) (Figure 4A). Sensitivity analysis revealed that multiple studies had an impact on the pooled results (Supplementary Figure 3A). Furthermore, no potential publication bias was detected (Begg's *p* = 1.00; Pustejovsky's *p* = 0.984) (Supplementary Figure 3B).

The analysis examined the relationship between infection and the occurrence of AKI. It was found that infected patients had a significantly higher incidence of AKI (OR: 3.60; 95% CI: 1.91, 6.78; *p* < 0.001) (Figure 4B). The sensitivity analysis confirmed the robustness of the pooled results (Supplementary Figure 4A). However, there was a possibility of publication bias (Begg's test *p* = 0.036; Egger's test *p* = 0.041) (Supplementary Figure 4B). And trim and fill method was employed to add potentially missing studies. After adjustments, the random effects model failed to detect a significant association between infection and AKI (OR: 2.12; 95% CI: 0.97, 4.64; *p* = 0.061).

The combined analysis revealed a significant association between comorbid hypertension and the incidence of incidence of AKI (OR: 4.02; 95% CI: 2.94, 5.51; *p* < 0.001) (Figure 4C). However, due to the limited number of studies included in the analysis, sensitivity analysis and publication bias analysis were not conducted.

**Figure 5:**
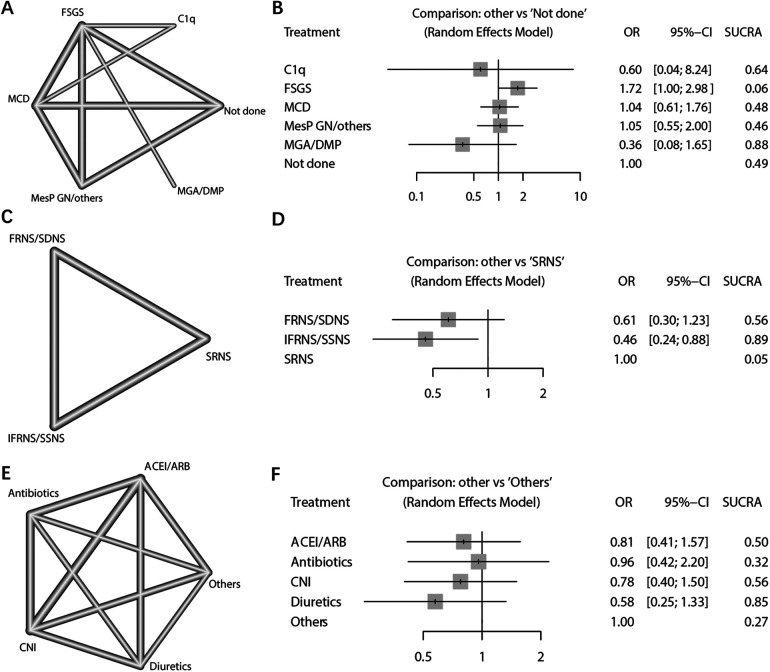
Network meta-analysis of NS pathological types, steroid-response NS types, and nephrotoxic drug types. **(A)** Network plot of pathological results; **(B)** Network Forest plot of pathological results; **(C)** Network plot of steroid response type results; **(D)** Network Forest plot of steroid response type results; **(E)** Network plot of types of nephrotoxic drugs; **(F)** Network Forest plot of types of nephrotoxic drugs.

In the analysis of NS characteristics, the risk of AKI was examined based on pathological findings and steroid sensitivity types. The pathological results included focal segmental glomerulosclerosis (FSGS), minimal change disease (MCD), minor glomerular abnormalities (MGA)/diffuse mesangial proliferation (DMP), C1q nephropathy (C1q), mesangial proliferative glomerulonephritis (MesPGN)/Others, and cases where pathological examination was not conducted (Figure 5A). Using the not done population as a reference, there was no significant difference in the risk of AKI among the other pathological types, except for the FSGS group. According to the SUCRA ranking, FSGS may be considered the pathological type with the highest risk of AKI (SUCRA = 0.07), while the pathological type MGA/DMP exhibited a relatively lower risk of AKI (SUCRA = 0.87) (Figure 5B).

**Figure 6:**
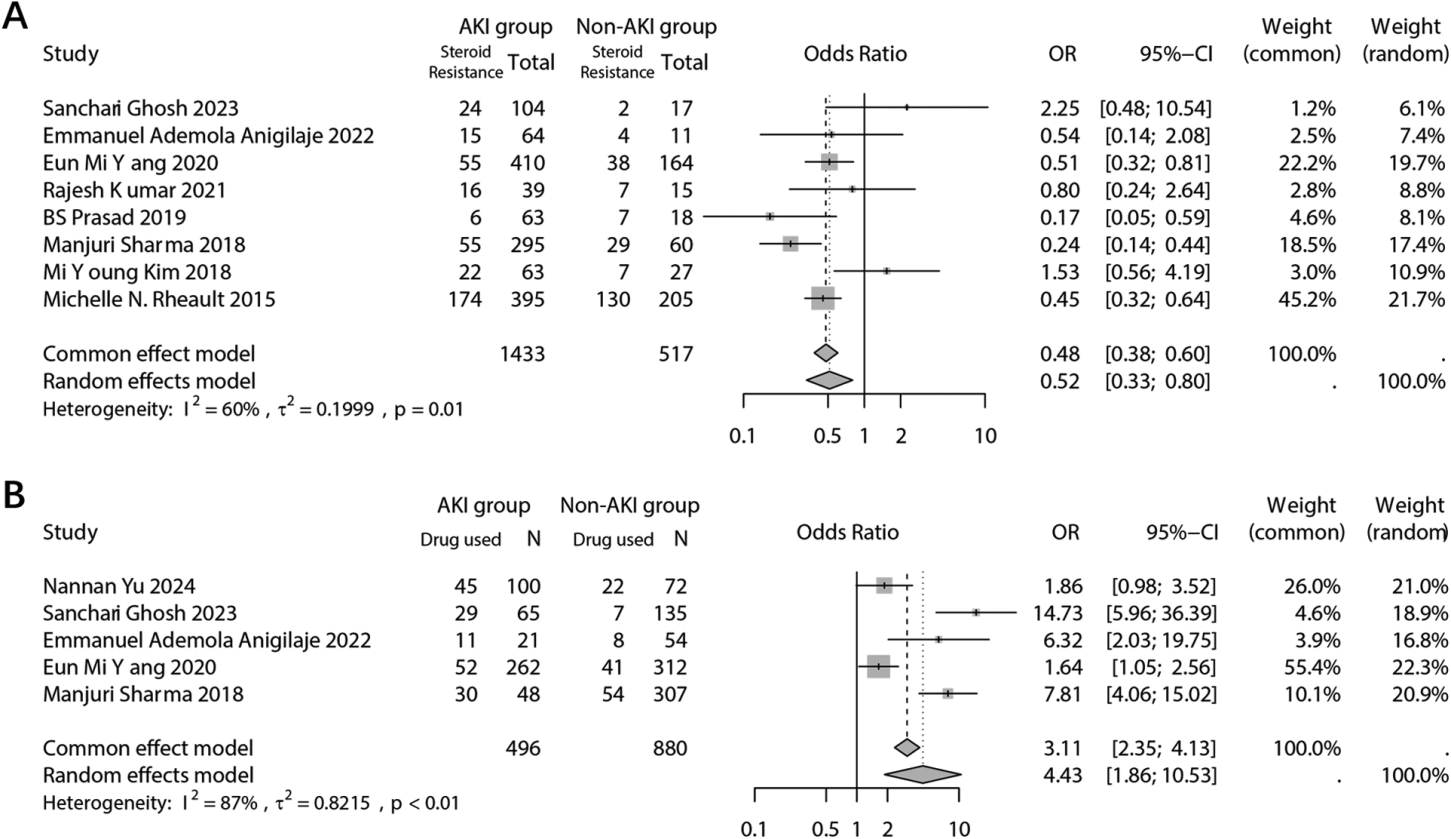
Forest plots showing the pooled results of steroid-resistance NS type **(A)** and use of nephrotoxic drugs **(B)** between the AKI group and the non-AKI group.

The analysis focused on the type of NS in response to steroid treatment. The dichotomous analysis revealed a higher incidence of AKI in individuals who exhibited resistance to steroid treatment (OR: 0.52; 95% CI: 0.33, 0.80; *p* = 0.003) (Figure 6A). Sensitivity analyses indicated that the study conducted by Michelle in 2015 had a significant impact on the final combined results (Supplementary Figure 5A). No evidence of potential publication bias was found (Begg's test *p* = 0.458; Egger's test *p* = 0.436) (Supplementary Figure 5B). Furthermore, the NS types were further subdivided into frequent relapses NS (FRNS)/steroid-dependent NS (SDNS), infrequent relapses (IFRNS)/steroid-sensitive NS (SSNS), and steroid-resistant NS (SRNS) (Figure 5C). When set SRNS as reference, the IFRNS/SSNS type was found to have a significantly lower incidence of AKI (OR: 0.46; 95% CI: 0.24, 0.88) and a higher SUCRA rank (0.89) (Figure 5D).

The combined analysis demonstrated a significant association between the use of nephrotoxic drugs and the incidence of AKI (OR: 4.43; 95% CI: 1.86, 10.53; *p* = 0.001) (Figure 6B). The sensitivity analysis confirmed the robustness of the combined outcome (Supplementary Figure 6A). Despite the inclusion of only five studies, a publication bias analysis was conducted. The results indicated no potential publication bias (Begg's test *p* = 0.142; Egger's test *p* = 0.149) (Supplementary Figure 6B). A network analysis was performed to examine different types of nephrotoxic drugs, including Angiotensin-Converting Enzyme Inhibitors (ACEI)/Angiotensin Receptor Blockers (ARB), antibiotics, calcineurin inhibitors (CNI), diuretics, and others (e.g., contrast agents, hypovolemia-inducing agents, ionotropes, etc.) (Figure 5E). When using the “others” arm as a reference, no other type of drug showed a significantly lower risk of AKI occurrence. Among the nephrotoxic drugs, diuretics exhibited a relatively high SUCRA score of 0.85, suggesting a relate low risk of AKI compared to other types of nephrotoxic drugs (Figure 5F).

## 4 Discussion

This study evaluated the incidence and risk factors for developing AKI in children with NS through a meta-analysis, firstly. The findings revealed that the overall risk of developing AKI in children with NS was 29%. The analysis of risk factors indicated that the age of NS onset, rather than the age of admission, sex, serum albumin level, response to steroid treatment, infection, hypertension, and nephrotoxic drug application, were all significantly associated with the incidence of AKI. Furthermore, the results of the network meta-analysis suggested that the pathologic type of MGA/DMP, the type of IFRNS/SSNS, and the use of diuretic medications were associated with a relatively low risk of AKI occurrence.

In the results of this study, the proportion of AKI following NS was higher in boys than in girls, and this proportion was statistically significant. Therefore, this conclusion is primarily drawn from the statistical findings of the population analysis. In terms of mechanism, sexual dimorphism exists in the gene expression of proximal tubules and endothelial cells in mice with ischemic AKI. This suggests that different sexes exhibit sex-specific gene expression patterns in response to ischemia. For instance, the upregulation of injury-associated genes lipocalin-2 (Lcn2), hepatitis A virus cellular receptor 1 (Havcr1), and keratin 18 (Krt18) is less pronounced in female proximal tubules compared to males. Conversely, adhesion molecules and cytokines/chemokines are upregulated in males but not in females (21). Additionally, variations in sex hormone production alter the susceptibility of different sexes to ischemic kidney injury (22). Estrogen may exert a protective effect in ischemic AKI, and this protective mechanism may involve gene targets such as estrogen sulfotransferase (SULT1E1/EST) and Sirtuin-3 (23, 24).

Children with NS often experience infections, which are a significant contributing factor to the development of AKI. Abnormalities in the immune system, characterized by a significant loss of immunoglobulins and complement regulators in the urine, increase the susceptibility to infections in NS patients. Furthermore, the use of immunosuppressive therapy further elevates the risk of infection. Following an infection, viruses and bacteria directly damage the renal tubular epithelial cells (25, 26). Additionally, the heightened systemic inflammation resulting from the infection, as indicated by elevated levels of interleukin-6 (IL-6) and tumor necrosis factor-alpha (TNF-α), has been found to correlate with increased levels of creatinine and urea nitrogen, thus being associated with the development of AKI (27). Inflammatory related factors such as soluble intercellular adhesion molecule-1 (ICAM-1), thrombomodulin, von Willebrand factor (vWF), and selectins have also been associated with the risk of AKI (28). Clinical studies have demonstrated that AKI is a common complication of sepsis, further highlighting the significant role of dysregulated inflammatory and immune responses in the development of AKI (29). The combined results of this study strongly suggest a substantial correlation between infection and the development of AKI in children with NS. However, it is important to note that the methodological analysis indicates the possibility of publication bias influencing the combined results.

Children with NS are more likely to be exposed to nephrotoxic drugs, and the risk of AKI increases with each additional nephrotoxic drug. The duration and intensity of nephrotoxic drug exposure are associated with the occurrence of AKI. Renin-angiotensin-aldosterone system (RAAS) inhibitors, for example, can cause vasodilation of small arteries and decrease intraglomerular pressure, leading to ischemia, increased cell membrane permeability, tubular injury, and deterioration of renal function (30). And then, the use of diuretics combined with hypovolemia due to urinary protein loss can further worsen renal tissue damage, leading to the development of thrombosis and AKI (31). The findings of this meta-analysis indicate a significant association between the use of nephrotoxic drugs and the occurrence of AKI. However, among the various types of nephrotoxic drugs, diuretics are relatively less likely to contribute to the occurrence of AKI compared to other nephrotoxic drugs. Nevertheless, caution should still be exercised when using diuretics, and they should only be administered after correcting the most severe forms of edema and hypovolemia (32). In conclusion, the administration of nephrotoxic drugs should be individualized based on the patient's glomerular filtration rate, with close monitoring of adverse drug reactions and regular assessment of renal function to minimize the risk of AKI.

Non-response to steroid therapy in children with NS is often indicative of a poor prognosis (33). In such cases, second-line immunosuppressive agents, primarily calcineurin inhibitors, are frequently employed. However, these drugs are considered nephrotoxic, which may contribute to the increased incidence of AKI. The progression of SRNS to multidrug-resistant NS can also lead to renal failure. Among cases of multidrug-resistant NS, FSGS is a commonly observed pathology type (34). Interestingly, the results of this study indicate that FSGS has the lowest SUCRA value among the pathology classifications, suggesting a relatively high risk of AKI development in these cases. The results of this study revealed a significant association between concomitant hypertension and an increased incidence of AKI.

A considerable proportion of children with NS exhibit hypertension (35, 36). Hypothyroidism, which can be observed in patients with NS, can contribute to hypertension due to the excessive excretion of thyroid hormones and binding globulins through the urine (37). Furthermore, hyperlipidemia, which can also lead to renal vein embolism, may be associated with the development of hypertension and AKI in NS patients (38). Moreover, medications such as prednisone, methylprednisolone, RAAS inhibitors, and CNIs have been implicated in raising blood pressure levels and increase the risk of developing hypertension (39). Taken together, these factors suggest a potential link between the use of immunosuppressive drugs, the severity of proteinuria, hypertension resulting from renal vein embolism, and the development of AKI. Further research is warranted to fully elucidate the underlying mechanisms and explore potential interventions to mitigate these risks.

The focus of this study was primarily on analyzing the association between relevant factors and the occurrence of AKI, rather than constructing risk prediction models. Specifically, the study aimed to identify differences between the AKI and non-AKI groups. Among the comparative results, *p*-values below 0.001 were observed for infection, use of nephrotoxic drugs, and hypertension. These factors can be given attention in the process of constructing a risk prediction model. On the other hand, risk factors such as age of NS onset, sex, and albumin level had *p*-values greater than 0.01. This suggests that their importance in the prediction model may be relatively lower.

This study has several research limitations that should be acknowledged. Firstly, there was a high degree of heterogeneity among the results of the included studies. This heterogeneity may have arisen from differences in the criteria used to assess AKI. Secondly, the design characteristics of the included studies were primarily prospective or retrospective cohort studies. These types of studies generally have a lower level of evidence compared to randomized controlled trials (RCTs). This is particularly relevant when examining the association between nephrotoxic drugs and the occurrence of AKI, as RCTs provide a higher level of evidence in establishing causal relationships. Furthermore, this study only explored the correlation between risk factors and the occurrence of AKI. Although, the selected risk factors were non-interventional, and there was a time lag between the recording of the risk factors and the occurrence of AKI (such as admission recording and AKI occurrence during hospitalization), this study is unable to draw definitive conclusions about the causal relationship between these risk factors and AKI. In fact, there may be intricate and intrinsic links between different risk factors, such as serum albumin level, infection, and hypertension, which could contribute to the development of AKI. Lastly, most of the studies included in this analysis were based on Asian populations and had relatively small sample sizes. Therefore, further well-designed studies with larger and more diverse populations are needed to validate and refine the results of this study.

## Data Availability

The original contributions presented in the study are included in the article/Supplementary Material, further inquiries can be directed to the corresponding author.
