# Supplementary material for: Incidence and risk factors for acute kidney injury in children with nephrotic syndrome: a meta-analysis
Source: Front Pediatr. 2024 Dec 20;12:1452568. doi: 10.3389/fped.2024.1452568 (PMC11695129; doi:10.3389/fped.2024.1452568)
Supplement: Supplementary file 1 [file Datasheet1.docx]

Supplementary Material

# Supplementary Figures and Tables

## Supplementary Figures

**
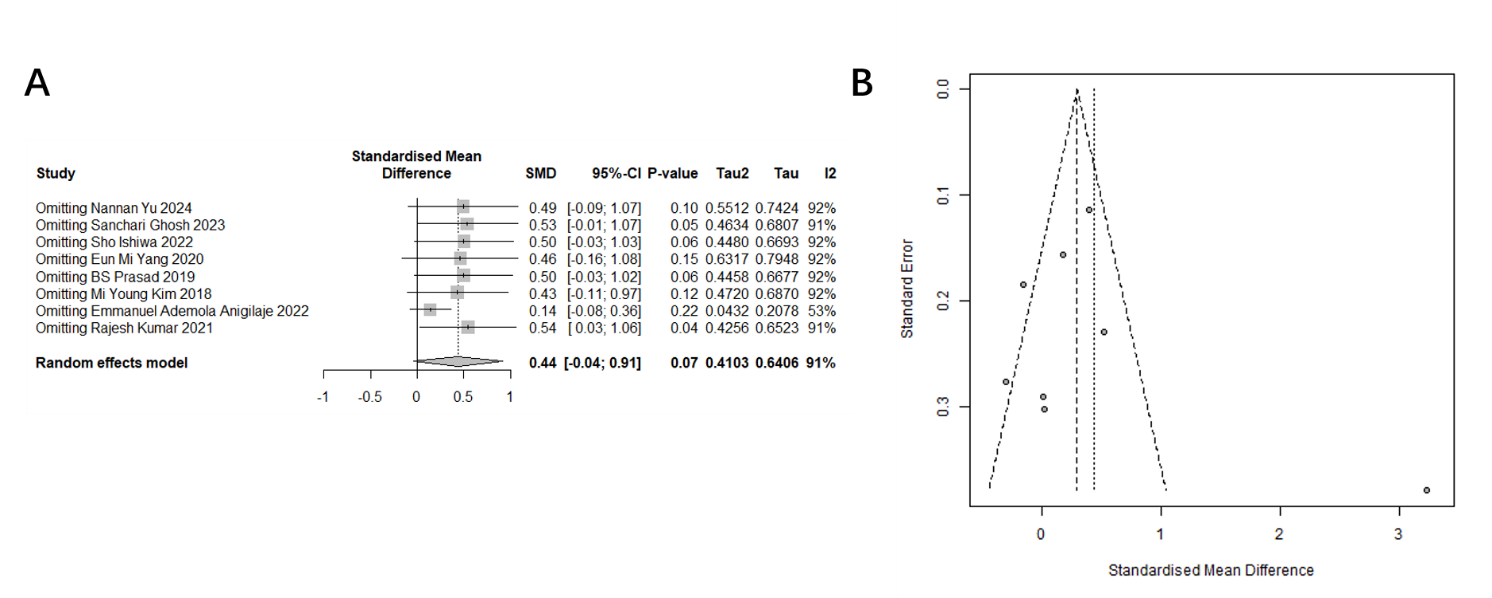
**

**Supplementary Figure 1.** Sensitivity analysis (A) and funnel plot (B) of the pooled analysis of age in admission between the AKI group and the non-AKI group.


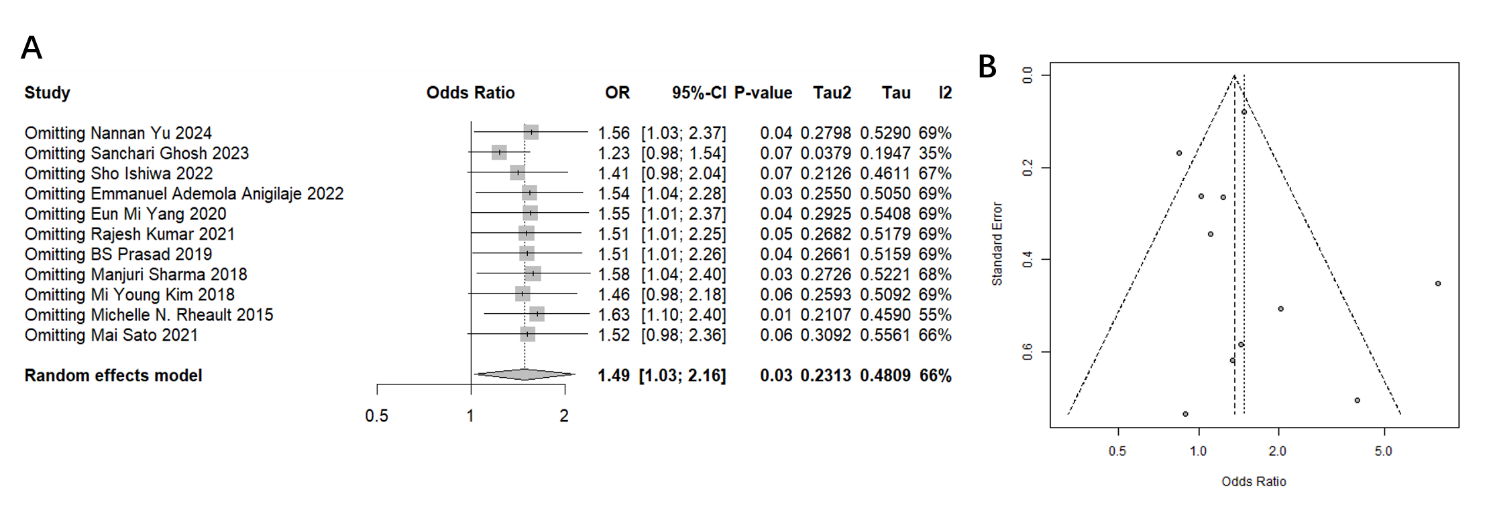


**Supplementary Figure. 2.** Sensitivity analysis (A) and funnel plot (B) of the pooled analysis of sex characteristics between the AKI group and the non-AKI group.


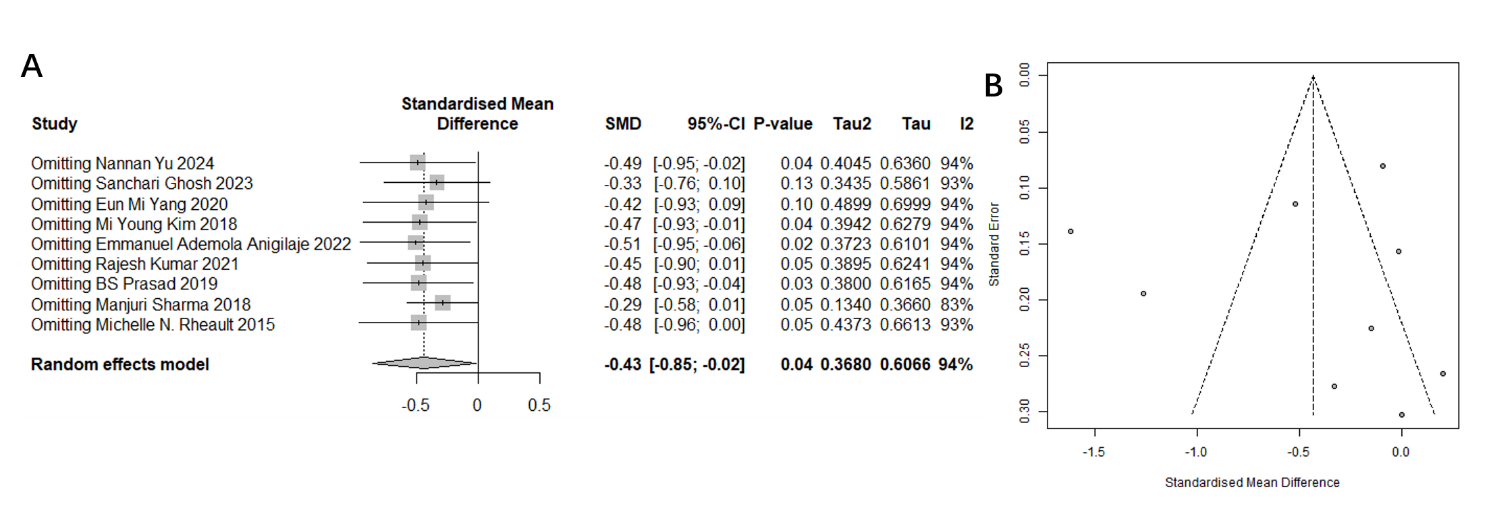


**Supplementary Figure 3.** Sensitivity analysis (A) and funnel plot (B) of the pooled analysis of serum albumin level between the AKI group and the non-AKI group.


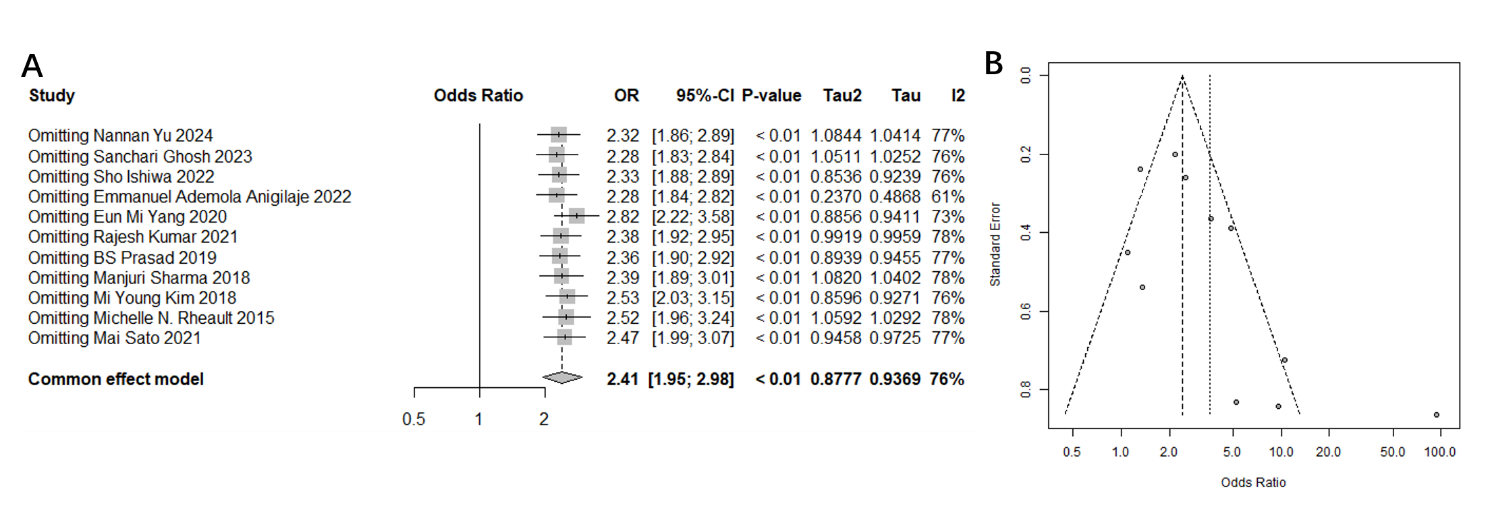


**Supplementary Figure 4.** Sensitivity analysis (A) and funnel plot (B) of the pooled analysis of infection rate between the AKI group and the non-AKI group.


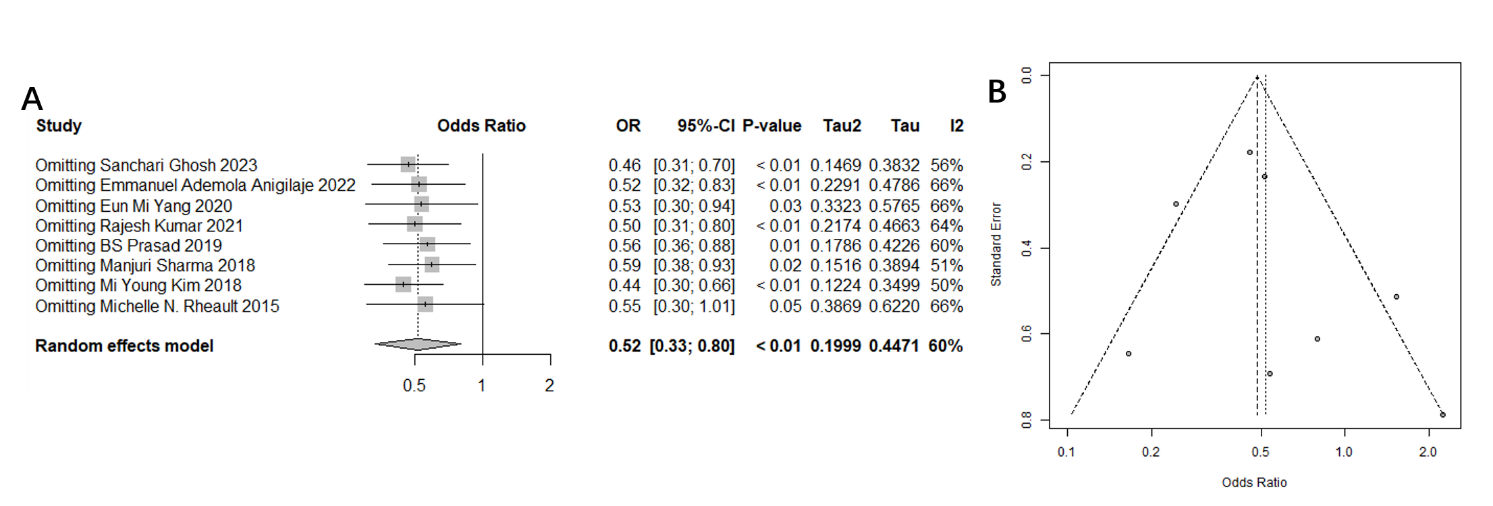


**Supplementary Figure 5.** Sensitivity analysis (A) and funnel plot (B) of the pooled analysis of steroid-resistance rate between the AKI group and the non-AKI group.


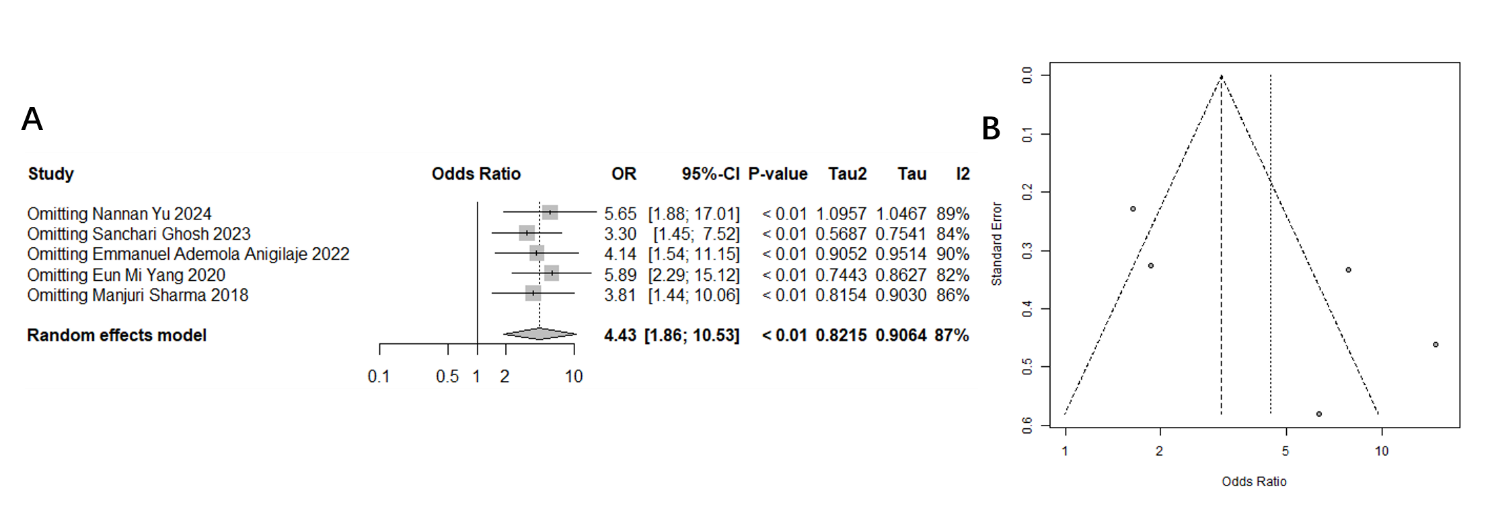


**Supplementary Figure 6.** Sensitivity analysis (A) and funnel plot (B) of the pooled analysis of use of nephrotoxic drugs between the AKI group and the non-AKI group.

## Supplementary Tables

Supplementary table 1. Design quality of included studies in detail

| Study | Representativeness of the Exposed Cohort | Selection of the Non-Exposed Cohort | Ascertainment of Exposure | Demonstration That Outcome of Interest Was Not Present at Start of Study | Comparability of Cohorts on the Basis of the Design or Analysis | Assessment of Outcome | Was Follow-Up Long Enough for Outcomes to Occur | Adequacy of Follow Up of Cohorts | Score |
| --- | --- | --- | --- | --- | --- | --- | --- | --- | --- |
| BS Prasad 2019 | 🟊 | 🟊 | 🟊 | 🟊 | 🟊🟊 | 🟊 | 🟊 | ☆ | 8 |
| Emmanuel Ademola Anigilaje 2022 | 🟊 | 🟊 | 🟊 | 🟊 | 🟊🟊 | 🟊 | 🟊 | 🟊 | 9 |
| Eun Mi Yang 2020 | 🟊 | 🟊 | 🟊 | 🟊 | 🟊🟊 | 🟊 | 🟊 | ☆ | 8 |
| Mai Sato 2021 | 🟊 | 🟊 | 🟊 | 🟊 | 🟊🟊 | ☆ | 🟊 | ☆ | 7 |
| Manjuri Sharma 2018 | 🟊 | 🟊 | 🟊 | 🟊 | 🟊🟊 | 🟊 | 🟊 | ☆ | 8 |
| Mi Young Kim 2018 | 🟊 | 🟊 | 🟊 | 🟊 | 🟊🟊 | 🟊 | 🟊 | ☆ | 8 |
| Michelle N. Rheault 2015 | 🟊 | 🟊 | 🟊 | 🟊 | 🟊🟊 | 🟊 | 🟊 | ☆ | 8 |
| Nannan Yu 2024 | 🟊 | 🟊 | 🟊 | 🟊 | 🟊🟊 | 🟊 | 🟊 | 🟊 | 9 |
| Rajesh Kumar 2021 | 🟊 | 🟊 | 🟊 | 🟊 | 🟊🟊 | 🟊 | 🟊 | 🟊 | 9 |
| Sanchari Ghosh 2023 | 🟊 | 🟊 | 🟊 | 🟊 | 🟊🟊 | 🟊 | 🟊 | 🟊 | 9 |
| Sho Ishiwa 2022 | 🟊 | 🟊 | 🟊 | 🟊 | 🟊🟊 | 🟊 | 🟊 | 🟊 | 9 |

Supplementary table 2. The subgroup analyses of AKI incidence across all subtype NS populations

| Subgroup | I^2^ | Rate | 95%CI |
| --- | --- | --- | --- |
| Sex |  |  |  |
| Boy | 88% | 31% | (24%, 40%) |
| Girl | 88% | 22% | (14%, 33%) |
| Pathological type | |  | (0%, 0%) |
| MCD | 85% | 34% | (22%, 53%) |
| FSGS | 68% | 45% | (31%, 64%) |
| MesPGN/oThers | 76% | 28% | (15%, 52%) |
| Not done | 96% | 30% | (12%, 73%) |
| MGA/DMP | - | 15% | (7%, 33%) |
| Hypertension | |  | (0%, 0%) |
| Yes | 0% | 39% | (31%, 49%) |
| No | 0% | 13% | (9%, 19%) |
| Infection |  |  | (0%, 0%) |
| Yes | 93% | 18% | (12%, 25%) |
| No | 84% | 44% | (33%, 59%) |
| Type of NS | |  | (0%, 0%) |
| IFRNS/SSNS | 88% | 22% | (13%, 38%) |
| FRNS/SDNS | 52% | 31% | (23%, 41%) |
| SRNS | 56% | 43% | (32%, 60%) |
| Nephrotoxic drug | |  | (0%, 0%) |
| Antibiotics | 0% | 49% | (36%, 68%) |
| CNI | 67% | 34% | (24%, 49%) |
| ACEI/ARB | 79% | 36% | (21%, 61%) |
| Diuretics | 73% | 28% | (15%, 53%) |
| Others | 85% | 42% | (18%, 100%) |

Abbreviations: AKI: acute kidney injury; CI: confidence interval; CNI: calcineurin inhibitors; DMP: diffuse mesangial proliferation; FRNS frequent relapses nephrotic syndrome; FSGS: focal segmental glomerulosclerosis; IFRNS: infrequent relapses nephrotic syndrome; MCD: minimal change disease; MesPGN: mesangial proliferative glomerulonephritis; MGA: minor glomerular abnormalities; NS: nephrotic syndrome; SDNS: steroid-dependent nephrotic syndrome; SRNS: steroid-resistant nephrotic syndrome; SSNS: steroid-sensitive nephrotic syndrome.
